# Indications, advantages and limitations of perinatal postmortem imaging in clinical practice

**DOI:** 10.1007/s00247-014-3165-z

**Published:** 2014-10-02

**Authors:** Owen J. Arthurs, Andrew M. Taylor, Neil J. Sebire

**Affiliations:** 1 Department of Radiology, Great Ormond Street Hospital for Children NHS Foundation Trust, Great Ormond Street, London, WC1N 3JH UK; 2 Institute of Child Health, University College London, London, UK; 3 Cardiorespiratory Division, Great Ormond Street Hospital for Children NHS Foundation Trust, London, UK; 4 Centre for Cardiovascular Imaging, UCL Institute of Cardiovascular Science, London, UK; 5 Department of Histopathology, Great Ormond Street Hospital for Children NHS Foundation Trust, London, UK

**Keywords:** Magnetic resonance imaging, Autopsy, Pathology, Foetus, Child

## Abstract

Just as there is a range of paediatric imaging techniques available during life, a similar repertoire is available as part of the foetal and perinatal postmortem examination. In this article, we review the literature regarding the diagnostic utility of postmortem radiography, US, CT and MRI in this clinical setting. There is limited direct evidence on the diagnostic utility of any of these techniques, apart from postmortem MRI, which when combined with other noninvasive investigations, has been shown to be highly sensitive and specific for many foetal postmortem diagnoses. The main disadvantages of postmortem MRI include the longer duration of imaging, the need for appropriate training in the interpretation of normal postmortem changes, and possible non-diagnostic imaging examinations in early gestation foetuses. As less-invasive autopsy becomes increasingly available, the true utility of these techniques will evolve, and clinical guidelines for maximal diagnostic yield can be developed.

## Introduction

In many developed countries, foetal and paediatric autopsy acceptance rates remain at historically low levels, with overall acceptance rates of about 12% in the United States and 15% in the United Kingdom [1, 2]. This low level of autopsy investigation means that large amounts of information that could be used to counsel parents about future pregnancies, contribute to epidemiological studies regarding foetal and infant deaths, and direct wider governance issues is currently not available. This decline is largely a result of reduced parental acceptance rather than clinical service provision, because in 80–90% of cases the clinicians discuss autopsy but the parents decline [3]. A combination of factors has led to this reduction, including parental reluctances and a corresponding desire for development of noninvasive and minimally invasive perinatal and paediatric autopsy service provision, including parental reluctance on moral or religious grounds, fear of disfigurement, delay in funeral plans, and lack of understanding the benefits [4]. This has led to a corresponding desire for the development of non-invasive and minimally invasive perinatal and paediatric autopsy services.

The ultimate role of autopsy is to determine the underlying cause and mechanisms of death, and in cases of stillbirth or foetal demise, to provide a unifying diagnosis that may have significance for the management of future pregnancies or implications for other family members. Obstetric US screening programs for early antenatal diagnosis have increased the frequency of terminations in early pregnancy during the last decades, and improvements in antenatal US imaging mean that there is now generally good agreement between prenatal US and autopsy findings, with 90% concordance in specialist centres [5]. This suggests that in many cases postmortem imaging is appropriate to confirm a foetal diagnosis, whether by radiography demonstrating a skeletal dysplasia or MRI demonstrating intracranial abnormalities. In certain circumstances there may be relatively little benefit in performing a full traditional autopsy.

Postmortem imaging adds value in three main ways. First, it provides a direct diagnosis, such as the radiographic phenotype in skeletal dysplasia. Second, it provides additional value to guide the autopsy, such as image-guided biopsy or identification of an unsuspected lesion. Third, in cases that parents do not agree to an invasive postmortem examination, postmortem imaging can be offered as at least some form of acceptable investigation after death, with the limitations of such an approach clearly defined. In discussing the perinatal postmortem imaging options in this article, we have drawn on the paediatric and adult literature where no perinatal evidence is available to discuss their relevance to perinatal postmortem imaging.

## Postmortem imaging modalities

**Fig. 1:**
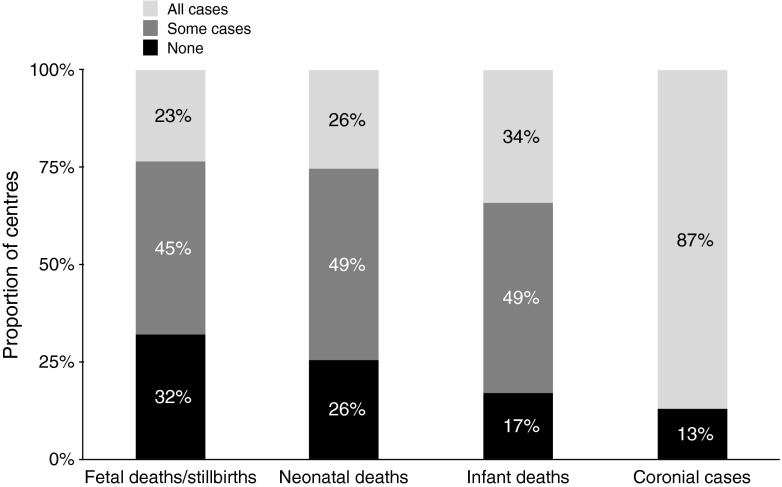
Consistency of postmortem imaging approach. Graph of the results from a survey of European Society of Paediatric Radiology members shows a lack of consistent approach regarding which sub-population of postmortem cases to image (foetal deaths, neonatal deaths, stillbirths or infant deaths). Reproduced with permission [6]

Several types of imaging can be employed in the investigation of foetal demise or termination of pregnancy including conventional postmortem radiography, US, CT and MRI. As might be expected, there are large differences in how these techniques are employed between institutions, across European countries and worldwide. A recent survey of European Society of Paediatric Radiology members reported that not only was there a lack of consistent approach regarding which sub-population of postmortem cases to image (foetal deaths, neonatal deaths, stillbirths or infant deaths) (Fig. 1), there was wide variation in the details of the services provided, such as imaging protocols used [6]. For instance, although the majority of postmortem imaging work in Europe is performed within diagnostic imaging departments by experienced paediatric radiologists with >5 years’ experience in postmortem imaging, the commonest modalities in current use are the postmortem radiography (80% of practitioners), postmortem CT (50%) and postmortem MRI (38%) [6]. Postmortem US was only offered at centres that also offered CT and MRI; only about half of centres had specific protocols for postmortem cross-sectional imaging, and there was significant variation among protocols based on little published evidence [6]. Less than a third of centres had standardised protocols for postmortem CT or postmortem MRI [6]. Multicentre international collaboration is required to establish standardised guidelines for perinatal and paediatric postmortem work to optimise service delivery and allow meaningful interpretation of data across centres.

### Foetal postmortem radiography

Skeletal radiography provides an overview of bone structure and development, and bone biometry, and it allows identification of focal or generalised bone abnormalities; a specific diagnostic approach to the postmortem radiography is provided elsewhere in this mini-symposium [7]. Routine postmortem radiographs are suggested in national autopsy guidelines and are considered mandatory in certain cases such as suspected skeletal dysplasia [8].

Before the widespread introduction of routine antenatal sonography, foetal gestational age estimation and accurate skeletal imaging was difficult. Postmortem radiography therefore developed as a standard tool for these indices from assessments of ossification centres and long-bone length measurements [9]. Postmortem radiography also allows rarer foetal disorders such as skeletal dysplasias to be detected and better characterised. However, postmortem radiographs can be technically difficult to perform because of the positional changes of rigor mortis (in infants and older children) and other artefactual issues, and unlike in live skeletal surveys, there is no standardised protocol or guidance as to when a babygram or skeletal survey should be used [10].

Modern antenatal sonography now provides highly accurate determination of gestational age and reliable detection of a wide variety of foetal abnormalities, including skeletal dysplasias. As antenatal diagnostic sonography continues to improve [5], combined with a low incidence and prevalence of skeletal dysplasias and other foetal bony abnormalities (the most significant of which are identified antenatally and are typically an indication for termination of pregnancy), the yield of *routine* postmortem radiographic examinations is low with regard to providing significant additional information in apparently anatomically normal foetuses and stillbirths. A particular challenge for radiologists in this setting is differentiating skeletal dysplasias from normal skeletal development at increasingly early gestation (less than 20 weeks), where knowledge of normal foetal appearances is crucial [7]. Other imaging modalities such as micro-CT and high-resolution postmortem MRI may become useful in these settings.

Skeletal abnormalities or dysplasias are extremely rare in infants who do not have external stigmata. This has been shown by studies in the 1970s and 1980s in which routine postmortem radiography was diagnostic in about 15% and of no value in >50% [11–13]. A more recent study suggested that postmortem radiography was not useful in the absence of an antenatal sonographic abnormality, abnormal clinical examination or abnormal chromosomal abnormality, although the authors did not address the utility of bone biometry for foetal growth assessment [14].

It is often difficult to extract from published data the objective *additional* value of postmortem radiography over an autopsy, or vice versa, because in all cases both investigations (autopsy and postmortem radiography) were performed and a composite final report issued. In studies where the autopsy rate was high, the additional yield of routine radiographs is likely to be minimal, with the main exception being specific clinical settings identified prior to autopsy, such as suspected skeletal dysplasias or complex genetic syndromes, in which postmortem radiography often provides a specific diagnosis. Two more recent studies of the objective usefulness of postmortem radiography have concluded that it is useful in only a minority of cases [15, 16]. The most recent study in a single specialist centre reported an overall abnormality rate of foetal postmortem radiography of about 10%, but almost all cases in this study had clear indications on antenatal history or external examination; only 0.3% of cases were predicted to be missed if a policy of highly selected, rather than routine, foetal postmortem radiography was employed [16].

Initial cost estimates suggest that a directed approach could also represent a cost saving: while postmortem radiography is fairly cheap as a diagnostic tool, the workload volume in routine imaging may not justify its overall service cost. An evidence-based selective postmortem radiography imaging protocol could yield significant cost savings, without a reduction in diagnostic yield. We conclude that routine postmortem radiography in all cases has a low diagnostic yield in clinical practice and is therefore neither diagnostically useful nor cost-effective, and therefore we suggest that postmortem radiography remain indicated for specific cases in which an appropriate abnormality is suspected from the antenatal findings, or in cases in which a specialist paediatric pathologist determines that there is a clinical indication to exclude or include specific features from the clinical history or initial external examination. Limiting skeletal radiography to selected cases would significantly reduce the radiology case workload. This would support the use of additional cross-sectional examinations such as postmortem CT in selected cases, yet still represent an overall cost-saving.

### Foetal postmortem sonography

Foetal sonography, although used widely in both antenatal and neonatal contexts, has, somewhat surprisingly, not been used or researched extensively in the postmortem setting. Postmortem US has been used in adults to demonstrate predominantly abdominal findings such as ascites, gallbladder stones, kidney and liver disease, and the presence of an intrauterine device. However normal postmortem changes in the body can cause difficulties at US, including hyperechoic abdominal and thoracic walls, gas distension of the digestive tract, and putrefaction of the subcutaneous tissues in infants [17]. Postmortem sonography is therefore better suited to foetal and perinatal postmortem imaging than paediatric postmortem imaging.

**Fig. 2:**
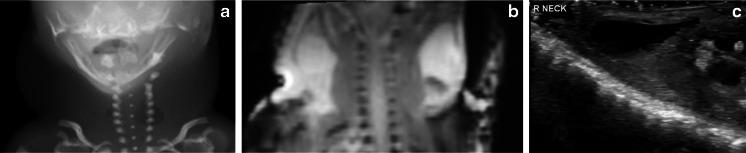
Cystic hygroma in female foetus of 14 weeks gestation. Hygroma was suspected on antenatal US scan (not shown), with the impression of a neck mass on postmortem radiograph (**a**). Postmortem MRI (**b**) was difficult to obtain because of size constraints, but postmortem US (**c**) was useful to delineate a bilateral cystic neck mass, which was confirmed to be cystic hygroma at autopsy

**Fig. 3:**
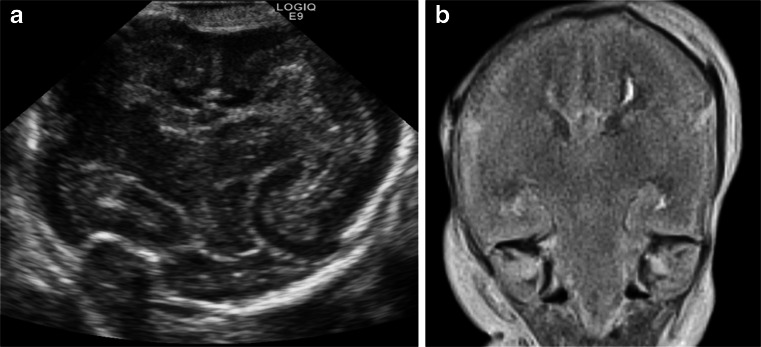
Postmortem US and MRI in a female foetus of 22 weeks gestation. **a** Cranial postmortem US. **b** Corresponding coronal T2-weighted postmortem MRI. Both show normal intracranial appearances. Note the minor deviation of the brainstem in the postmortem US image, caused by positioning

A skilled sonographer can obtain excellent imaging of the cranial contents, spine, limbs, chest and abdomen, and at our institution sonography has been found to be particularly useful in two settings: when the foetus is too small to be reliably imaged with routine postmortem MRI (typically in the 12- to 16-week gestation age range), and to address a specific issue raised at postmortem radiography or MRI. Examples include suspected ray abnormalities in the hand that cannot be resolved using plain radiographs, or abnormalities such as cystic hygroma in a small foetus (Fig. 2). Cranial sonography is technically familiar to the paediatric radiologist and would be easily performed in the postmortem setting, but there is only anecdotal evidence for its use thus far (Fig. 3).

Compared to antenatal US examination, postmortem US has several significant potential advantages for achieving optimal imaging: the body can be positioned in an optimal orientation with no movement, and the high-frequency probes can be used in close proximity to the target tissue. However, both pliability and postmortem changes within tissues can pose a problem in accurate localisation of lesions in postmortem US in foetuses, and rigor mortis can present further adaptation problems. Postmortem US also requires the operator to be in prolonged direct contact with the body, which may inhibit the widespread uptake of this technique by practitioners. With relatively inexpensive and easy-to-acquire hardware, postmortem US has the potential to contribute greatly to postmortem imaging in this setting but further research is required to determine optimal methods and approaches.

Although percutaneous organ biopsy using surface landmarks has been attempted [18, 19], US-guided percutaneous biopsy has not been studied in detail in foetuses. Neonatal percutaneous biopsy samples do not always yield adequate tissue volumes [18], and when they do so they may still be non-diagnostic [19]. As such, although US-guided percutaneous biopsy may be an attractive method of noninvasive tissue sampling, and it shows good success rates in older children [20], there may be technical limitations in foetuses and stillbirths including small size, accuracy and postmortem artefacts making organ differentiation difficult. With continuing improvements in imaging and experience, image-guided biopsy may become more important when fully developed.

### Foetal postmortem CT

The main advantages of postmortem CT over postmortem MRI are speed, availability and the increased bone detail that is achieved in CT. Although postmortem CT angiography is now the mainstay of adult postmortem imaging [21, 22] to demonstrate coronary artery disease and vascular pathology throughout the body that could relate to the cause of death, both contrast-enhanced and non-contrast-enhanced postmortem CT present difficulties in imaging foetuses and children, making its use less appropriate.

**Fig. 4:**
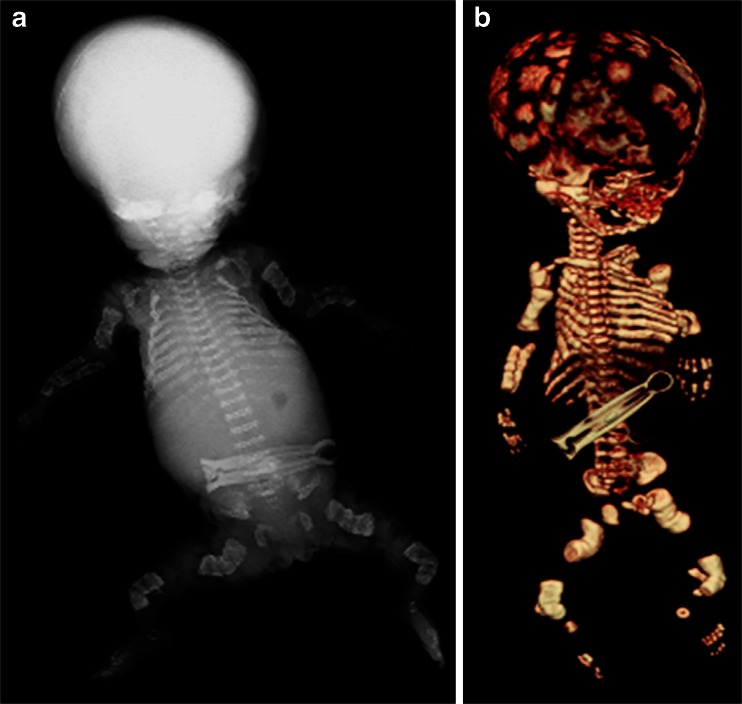
Postmortem imaging in a male fetus of 20 weeks gestation suspected of having skeletal dysplasia. Both postmortem radiograph (**a**) and postmortem CT image (**b**) of a foetus terminated for suspected skeletal dysplasia show crumpled long bones and ribs, representing multiple fractures of osteogenesis imperfecta type II. Image (**a**) reproduced with permission [16]

In the context of foetal and childhood pathology, unenhanced postmortem CT gives excellent bony detail and provides good diagnostic-quality images in suspected skeletal dysplasias, with possible additional benefits from 3-D reconstructions (Fig. 4), and in fracture imaging in suspected neonatal non-accidental injury. However, it is difficult based on available data to quantify any diagnostic advantages of postmortem CT over plain radiography for dysplasia imaging [23], and no robust studies show a diagnostic advantage of postmortem CT over plain radiography for fracture imaging. It may be that bone length/biometry, ossification centres and the developmental stage of deciduous teeth are easier to determine on postmortem CT images than on postmortem radiographs, although only small studies have been carried out to date [24].

One study has suggested that postmortem CT can be useful in selected cases; in a small cohort of 47 deaths in infancy, good concordance between postmortem CT and autopsy findings was reported (89%; 95% confidence intervals, 77.4–95.4%) although deaths remained unexplained in 29 of their 47 cases (62%) [25]. Other preliminary studies have used postmortem CT to exclude child abuse (bony injuries) in children, but with minimal positive findings. Oyake et al. [26] wrote that “it was difficult to presume the cause of death with [postmortem] CT alone” and that laboratory data and microbiology were required for most of the diagnoses encountered. Postmortem CT may be useful in traumatic deaths, particularly in head injury and in the evaluation of suspicious deaths or non-accidental injury, but that is outside the scope of this article.

**Fig. 5:**
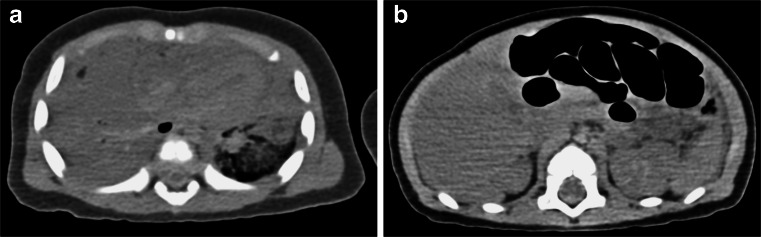
Unenhanced postmortem CT in a 14 day old female infant who died unexpectedly, the cause of death was unascertained. Unenhanced postmortem axial CT of the chest (**a**) and abdomen (**b**) in a neonate demonstrate poor signal contrast, with limited differentiation between heart and non-aerated lungs, and abdominal organs

**Fig. 6:**
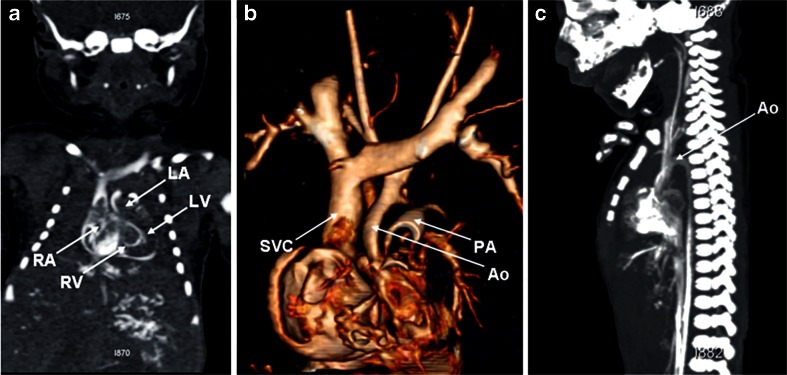
Postmortem CT angiography of the heart in a 37-week foetus with normal cardiac anatomy. **a** A four-chamber view, (**b**) reconstructed 3-D view of the great vessels, and (**c**) aortic arch are demonstrated. *Ao* aorta, *LA* left atrium, *LV* left ventricle, *PA* pulmonary artery, *RA* right atrium, *RV* right ventricle, *SVC* superior vena cava. Reproduced with permission [29]

The main disadvantages of postmortem CT compared to postmortem MR in foetuses and children include (1) markedly inferior soft-tissue contrast from reduced abdominal and subcutaneous fat, and (2) the lack of intravenous contrast agent, which makes assessment of the thoracic and abdominal cavity organs almost impossible (Fig. 5). Although postmortem CT angiography is gaining popularity for adult postmortem imaging, with intravenous contrast agent administered via femoral access [27, 28], the application of intravenous contrast agent via the femoral vessels in tiny foetuses is technically difficult, although the umbilical vein can be used because it is more accessible. The only reproducible way of imaging the heart in late-gestation foetuses is via direct intracardiac contrast injection [29] (Fig. 6). A diagnostic accuracy study of postmortem CT angiography versus postmortem MRI for congenital cardiac disease remains to be conducted, but given the noninvasiveness of postmortem MRI, this modality is likely to be preferred by parents.

Micro-CT is a potential alternative diagnostic modality for imaging small bodies, using CT but at improved resolution down to micrometers rather than millimetres. Micro-CT is becoming more widely used in postmortem forensic work [30] and has recently been used to image foetal hearts [31], although extracting and fixing tissue for optimal contrast is necessary.

### Foetal postmortem MRI

Foetal postmortem MRI shows the greatest promise as an adjunct and possible alternative to conventional perinatal and paediatric autopsy. Many advances have been made since several early postmortem MR studies were published in the late 1990s suggesting reasonable sensitivity and specificity for brain and spinal cord abnormalities [32–34]. More recent studies have also suggested that postmortem MRI can be used to perform other functions, usually during autopsy, such as organ weight or volume estimation [35–37].

**Fig. 7:**
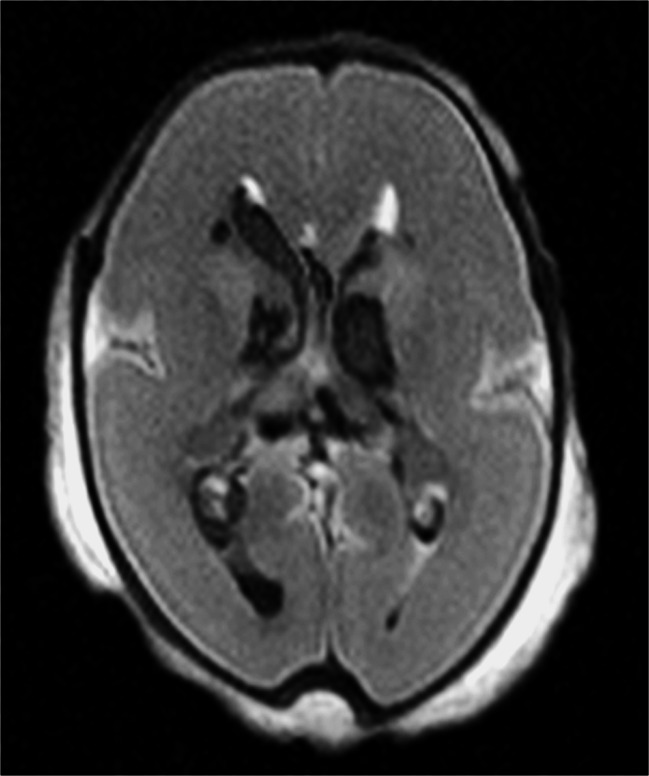
Postmortem MRI of the foetal brain. Axial T_2_-weighted postmortem MR image of the brain in a 23-week foetus shows bilateral intraventricular and periventricular haemorrhage

**Fig. 8:**
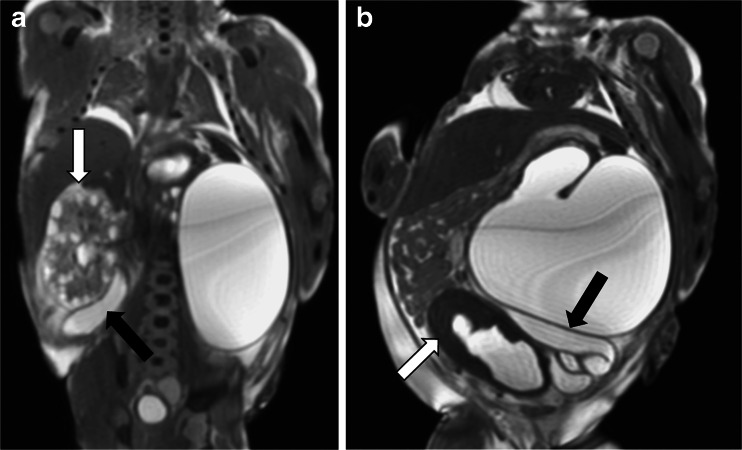
Postmortem MRI of the abdomen in a female fetus of 22 weeks gestation. Coronal (**a**) and parasagittal (**b**) T_2_-weighted postmortem MR images in a late stillbirth with obstructive uropathy. The right kidney is multicystic dysplastic (*white arrow* in **a**) and the left kidney is obstructed, with gross tortuous dilatation of both ureters (*black arrows*), and a thickened trabeculated bladder (*white arrow* in **b**), representing bladder outflow obstruction. Secondary pulmonary hypoplasia is also demonstrated. Posterior urethral valves were confirmed at autopsy

The largest recent prospective trial of postmortem MRI versus standard traditional autopsy in foetuses, stillbirths and children showed that postmortem MRI had the highest diagnostic accuracy in the foetal age group (Magnetic Resonance Imaging in Autopsy: MARIAS study) [38]. This study, which included 277 unselected foetuses out of a total 400 cases, reported the greatest concordance between conventional autopsy and less-invasive autopsy (defined as postmortem MRI including ancillary investigations such as examination of the placenta, but no invasive incisions) of 94.6% for foetuses <24 weeks, 95.7% for foetuses >24 weeks, compared to 76.4% in children [38]. Furthermore, postmortem MRI was particularly accurate at identifying brain, cardiac and renal pathologies (Figs. 7 and 8), as might be expected from in vivo experience, but postmortem MRI was poorer at defining intestinal pathology or lung pathology, such as infection.

Postmortem MRI alone did not detect about one-quarter of all major foetal diagnoses, cases in which additional genetic or placental examination was required, or detect cases of sepsis [38]. This comprehensive study demonstrated the importance of postmortem MRI as a component of a less-invasive autopsy examination that includes the clinical history, detailed external examination, skeletal radiographs (where indicated), placental analysis, and other ancillary investigations as appropriate [38]. It also showed that the combination of an experienced perinatal pathologist and radiologist could predict with high accuracy (>99%) which cases would require additional full or selective autopsy following the postmortem MRI and other less-invasive autopsy investigations [38]. Further analysis of these and other data should allow preliminary clinical guidelines to be established, detailing which foetuses (stillbirths, terminations, suspected syndromic diagnoses) would most likely benefit from which imaging approach.

However, foetal postmortem MR cannot be approached and introduced without appropriate safeguards. An understanding of the imaging correlates of normal postmortem changes such as fluid redistribution (subcutaneous oedema, pleural and pericardial effusions and ascites) remains elusive and the subject of ongoing research. Similarly, reliably discriminating pathological processes from normal postmortem changes on imaging alone is particularly difficult in the lungs and the abdomen. Foetal imaging is further complicated by the potential superimposed effects of maceration following in utero death, and subsequent postmortem interval-related changes, which vary with gestational age. A comprehensive review of the normal findings on postmortem MR is also included elsewhere in this issue [39].

With the increasing introduction of routine first-trimester antenatal US screening, foetuses may be submitted for autopsy examination at early gestational ages. These represent a challenge, both for traditional autopsy examination and imaging approaches, and in certain instances postmortem imaging is useful where the autopsy is non-diagnostic. However, in the MARIAS study [38] postmortem MRI was non-diagnostic in about a third of foetuses younger than 24 weeks’ gestation. Several imaging approaches are possible in very early gestation foetuses. The use of postmortem sonography has been discussed and may provide additional useful information but current data are lacking and this approach is likely to be useful for specific abnormalities only. Very-high-field postmortem MRI has been described and initial diagnostic feasibility demonstrated. In a study of foetuses <22 weeks’ gestation imaged at 1.5 T and 9.4 T, conventional MRI at 1.5 T was non-diagnostic in 14/18 cases, but MRI at 9.4 T as well as autopsy were diagnostic in all 18 cases [40]. However, high-field MR scanning capability is not widely available and remains predominantly a research tool, although a case could be made for centralising services for precisely this provision.

## Role of postmortem imaging across clinical settings

In some circumstances postmortem MR might be superior to traditional autopsy and might provide diagnostic information over and above that achieved at standard open autopsy. In the MARIAS study [38], postmortem MRI enabled detection of clinically significant lesions in about a third of cases in a subgroup of foetuses in whom formal neuropathological examination was inconclusive because of autolysis and other postmortem changes. This suggests that in foetuses with suspected neuropathological abnormality, routine pre-autopsy cerebral MRI might be a pragmatic step in case the neuropathology is uninformative. Nevertheless, even in this setting normal changes after death can make interpretation on MRI findings difficult. For example, in a study of foetuses with antenatally diagnosed cerebral ventriculomegaly, many of whom also had confirmation on foetal (in utero) MRI examination, postmortem MRI demonstrated resolution of ventriculomegaly prior to autopsy in about half of cases [41].

Postmortem imaging may provide specific information to allow targeted tissue sampling or direct endoscopic assisted sampling in cases in whom parents do not agree to traditional autopsy. In cases where the parents do not consent to any form of invasive investigation, postmortem MR can, of course, provide more information than no examination at all. The varied clinical circumstances in which postmortem MR may be requested must be appreciated when attempting to determine its value, because this differs according to the specific clinical question to be addressed and the availability or unavailability of other forms of postmortem investigation in any individual case.

## Can a normal perinatal postmortem examination be helpful?

Just as MRI now offers an additional step in the antenatal diagnostic imaging pathway, perinatal postmortem imaging has two significant indications: (1) to confirm that antenatal findings were correct (where an abnormality is suspected), and (2) to confirm the absence of any other abnormality missed on antenatal scanning. For instance, a foetus terminated for apparently isolated ventriculomegaly on antenatal scanning in whom no other abnormality is identified on postmortem imaging or autopsy represents a different process compared to one in whom additional findings are present to suggest a specific underlying syndromic diagnosis. In the former, where the findings are confirmed, the parents may be reassured regarding the antenatal findings and this provides an important governance tool. It is difficult to quantify the effect that a normal finding may have on grieving parents during a future pregnancy, but this role should not be underestimated.

By virtue of the population being assessed, and the evidence from the large cohort trials already performed, the majority of imaging and autopsy investigations by whatever means are likely to be normal. Can we quantify how useful a normal postmortem imaging study could be? The majority of postmortem radiographic and postmortem MR studies have a high negative predictive value, which suggests that when imaging is normal few pathologies are missed. This can be highly reassuring to both the pathologist and parents, particularly when further invasive autopsy is declined. However, there are two other implications to a normal study in which no significant abnormality was identified.

First, a failure to find an abnormality in the foetus following intrauterine death, stillbirth or miscarriage potentially implicates a greater likelihood of a maternal–placental unit pathology as the underlying cause. For example, an unexpected late stillbirth with normal antenatal history and normal antenatal US examinations, who then has a normal postmortem MR, could help to direct the pathologist to assess the placenta and the obstetrician to investigate other maternal abnormalities. The approach to the autopsy may also be significantly modified if a specific abnormality is suspected on postmortem imaging, such as a brain or cardiac abnormality. This can therefore be useful to direct resource allocation.

Second, a normal postmortem imaging investigation represents a cohort of foetuses in whom no specific anatomical diagnosis is present, in whom future studies investigating other specific ancillary investigations can be targeted, for example cardiac arrhythmias or genetic studies [42, 43], representing a triage step in designing future studies.

## Minimally invasive autopsy sampling

There is one specific indication for which postmortem MRI, with or without other imaging modalities, represents an essential and integral part of the process, namely the minimally invasive autopsy. A minimally invasive autopsy is based on postmortem imaging followed by targeted tissue examination using a variety of techniques including endoscopic guidance. This process allows similar clinically relevant diagnostic information to be obtained compared to standard traditional autopsy for selected indications in whom the parents do not consent to a standard traditional autopsy approach. Because neither selected tissue biopsy nor endoscopic examination and sampling allow adequate anatomical information to be obtained, it is essential that postmortem MR is carried out beforehand. Finding either a normal or specifically abnormal anatomical examination is extremely useful to direct further investigation, to allow the maximum diagnostic yield with the least invasive approach. It should also be recognised that in some cases parents will not agree to any form of autopsy tissue sampling, but in these cases postmortem imaging combined with ancillary investigations, such as placental histological examination, can still provide useful clinical information. Initial studies indicate that postmortem imaging using MRI is a highly acceptable approach even to those who do not agree to a standard autopsy and, furthermore, the minimally invasive autopsy appears more acceptable than the standard open procedure; it is therefore highly likely that these approaches will become more widespread [44–47].

## What will a future service look like?

**Fig. 9:**
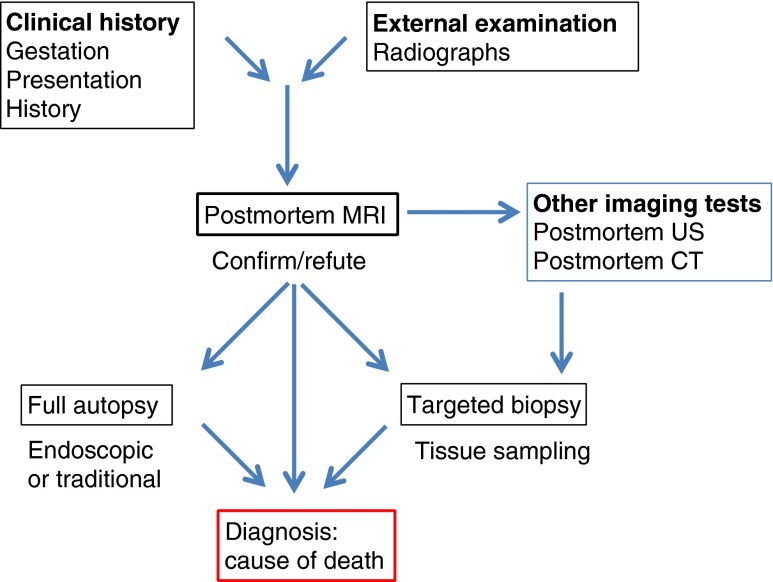
Perinatal postmortem imaging service approach. Diagram illustrates a speculative outline of a stepwise approach to incorporating postmortem imaging into a comprehensive perinatal postmortem service

The integrated foetal postmortem imaging service will include a multidisciplinary group of obstetricians, foetal medicine specialists, paediatric radiologists, perinatal pathologists, and geneticists, to name but a few, who might be involved in the ongoing care and counselling of bereaved parents. By combining these clinical skill sets and recognising the contribution of each imaging and other modality to the final diagnosis, the optimal approach to the investigation after death can be determined for each case. We consider that following foetal death, a stepwise approach is the most logical and efficient, using, where available, a full clinical history and examination that take into account gestation, presentation and likely diagnosis (Fig. 9). Skeletal radiographs are to be performed where clinically indicated, followed by a postmortem MRI in all cases in whom it may direct a full standard autopsy or in whom the parents decline traditional autopsy examination. Postmortem CT and postmortem US should be used to address specific issues, and on the basis of all the imaging (antenatal and postmortem), targeted biopsy or full autopsy can be performed.

## Conclusion

The use of postmortem imaging in foetal and paediatric diagnosis will continue to evolve and improve. We suggest that a targeted, rather than routine, radiographic approach and more routine postmortem MRI approach will give the highest diagnostic yield, particularly in cases in which a formal autopsy is declined. By understanding the advantages and limitations of each imaging technique, we can employ each technique to its maximal advantage and counsel parents appropriately as to what a normal scan means in the appropriate context. As minimally invasive autopsy becomes increasingly available, the true utility of these techniques will become clear and help to develop clinical guidelines for maximal diagnostic yield and parental acceptability of the investigation after death.
